# Editorial: The Bacterial Cell: Coupling between Growth, Nucleoid Replication, Cell Division, and Shape

**DOI:** 10.3389/fmicb.2016.00116

**Published:** 2016-02-15

**Authors:** Jaan Männik, Conrad L. Woldringh, Arieh Zaritsky

**Affiliations:** ^1^Department of Physics and Astronomy, Department of Biochemistry and Molecular and Cellular Biology, University of TennesseeKnoxville, TN, USA; ^2^Bacterial Cell Biology, Faculty of Science, Swammerdam Institute for Life Sciences, University of AmsterdamAmsterdam, Netherlands; ^3^Faculty of Natural Sciences, Ben-Gurion University of the NegevBe'er-Sheva, Israel

**Keywords:** chromosome replication, nucleoid, cell division, divisome, envelope, cell shape, *Escherichia coli*, cell cycle

## Introduction

The nucleoid and cell envelope are two unique macromolecules that define a bacterium. To ensure that the essential genetic information is carried faithfully from one generation to the next their duplications must be coupled. Research dating back more than half a century established that this is realized at the level of replication-initiation and cell division. These seminal investigations demonstrated that cell size determines when replication starts and that, in turn, the progress in replication determines when cell division processes occur. Moreover, cell division was later established to be spatially controlled by the structure and position of the nucleoid. Despite extensive research over the past 60 years, detailed understanding on how these spatio-temporal couplings are realized at the molecular level is yet to emerge. The aim in creating this e-book has been to bring together scientists from different backgrounds spanning biology, chemistry and physics to address these fundamental mechanisms in a bacterial cell. The book starts with a historical overview of the research that has established the field. It then advances through series of reviews and research articles devoted to the nucleoid, cell envelope, and their temporary hyper-structures, the orisome, replisome, and divisome. Finally, coupling between them is discussed in the last set of contributions.

## Historical overview

The first evidence of coupling between duplications of the bacterial cell envelope and the nucleoid was revealed in the 1950s and 1960s. Milestones in this research were demonstrations of replication initiation at a constant cell mass per *oriC* and constant *C* and *D*, dissociated from growth dynamics, where *C* denotes replication time and *D* time from replication–termination to the separation between the subsequent daughter cells. These findings form the basis of our understanding of physiology of bacterial cell duplication and guide the current research. Historical accounts of how these ideas came about are covered in the e-book by the scientists who were instrumental in developing these concepts (Hanawalt; Helmstetter; Schaechter; Zaritsky and Woldringh). The articles dwell on scientific achievements and personal experiences, which endows the new generation of scientists valuable perspective.

## The nucleoid

The nucleoid as a central macromolecular complex of bacterial cell harbors several hyper-structures that are essential to its organization and coupling to the envelope. These hyperstructures include orisomes, replisomes, transcriptional assemblies, and transertional linkages that connect DNA through transcribed mRNA and coupled translated membrane protein to the plasma membrane of the cell. Leonard and Grimwade describe how a timing mechanism, based on unwinding of *oriC* by the highly conserved DnaA, starts the orisome assembly. Next, the composition and architecture of the replisome is discussed with emphasis on dynamics and stability during its “journey” from origin to terminus (Beattie and Reyes-Lamothe). Despite detailed knowledge of the orisome and replisome components from both *in vivo* and *in vitro* studies, we have little understanding of how these structures are spatially correlated and physically positioned in the cell. Regarding this aspect, Woldringh et al. present new data on movement and positioning of fluorescently tagged chromosome arms in slow-growing *Escherichia coli* cells. Remarkably, the observed segregation patterns were similar to those obtained during run-off DNA replication in rifampicin-inhibited cells, suggesting that segregation is passively driven by *de novo* DNA synthesis.

Analysis of super-resolution fluorescence images of ribosomes and RNA polymerase in rapidly growing *E. coli* cells enabled Bakshi et al. to propose a “translation-centric” view of the compartmentalization of cytoplasm and nucleoid. Their observations are supported by time-dependent effects of rifampicin and chloramphenicol inhibition on nucleoid-ribosome morphology and could be simulated by a physical model assuming entropic contributions and excluded volume interactions between ribosomal subunits and DNA. In a more “transcription-centric” view of nucleoid morphology, Jin et al. describe how multiple ribosomal RNA operons form a nucleolus-like structure at the periphery of the nucleoid.

How these cytological observations of nucleoid behavior can be understood in a more quantitative, physical perspective remains to be seen. For instance, following a well-known concept in material science, Scolari et al. provoke us to think of the bacterial nucleoid as a smart polymer—a gel-like substance that shows a variety of responsive behaviors to external stimuli such as temperature, pH, and ionic strength.

Coupling between mass growth and chromosome replication has been studied for six decades using mutants defective in thymine biosynthesis (Guzmán and Martín). Yet, what leads to thymineless death and whether this process is actively controlled are still open questions (Ostrer et al).

## Cell envelope and shape

Different bacteria display different cell shapes in reaction to environmental changes, including light-dependent responses (Montgomery). The underlying shape changes depend on the peptidoglycan synthesizing machinery, which is largely conserved and modular as reviewed by Randich and Brun. On the other hand, as emphasized by De Pedro and Cava, the structural diversity of the peptidoglycan wall is larger than expected on the basis of *E. coli* data. An intriguing hypothesis that tries to connect the structure and organization of the nucleoid with growth of the cell envelope is the transertion mechanism, i.e., coupled transcription, translation, and insertion of membrane proteins. Matsumoto et al. review how transertion may induce heterogeneity of lipids and proteins in the membrane that might ultimately influence local peptidoglycan synthesis leading to cell division.

## The divisome

Life requires cell division. Norris argues that division is essential to maintain the connectivity of cellular components. However, some bacteria can grow surprisingly large and apparently still retain all their essential functions except division (El-Hajj and Newman). One of the biggest, *Epulopiscium fishelsoni*, clearly defies limits expected to be set by diffusive transport.

On a molecular level, the divisome is a busy factory that involves over 30 proteins in *E. coli*. How these proteins come together and form this hyper-structure is still unclear. Here, both Egan and Vollmer, and Vischer et al. address the stoichiometry of the divisome components. New interactions among the components are reported in three research articles (Gamba et al; Lies et al; Sieger and Bramkamp), and a thorough review of new fluorescent probes for labeling the divisome is presented by Wu et al.

## Spatio-temporal coupling mechanisms

The last set of articles in the e-book investigates spatio-temporal coupling between the nucleoid, divisome, and cell size. The divisome positioning in *E. coli* is realized by two partially redundant mechanisms that use chromosome as a structural scaffold (Männik and Bailey) but also in an apparently chromosome-independent manner by the Min proteins (Rowlett and Margolin). None of these molecular systems is essential provided that cell division occurs sufficiently infrequently (Männik and Bailey). The bacteria may be even capable of propagating without any spatial coupling between the nucleoids and the divisome although at the expense of much lower fitness.

Seminal studies in the 1960s established a relationship between initiation of replication and cell size using population average measurements. A series of recent analyzes of large numbers of individual cells have shown that they grow by adding a constant length/volume from one division to the next. Taheri-Araghi argues that an incremental growth model is consistent with the old ideas of initiation at a constant cell mass per *oriC* and finds a consistency between the model and experiment in an analysis that assumes invariant *C* and *D* periods for all cells. The new findings also raise the question of how a cell senses that it has added a constant length increment since its birth. Ho and Amir propose that the size increment is realized by constant accumulation of replication initiator factors. When the concentration of these factors reaches a threshold they trigger new round of replication. The authors assume that the initiation factors are degraded immediately after start of replication and that their accumulation then starts anew. The model accounts for experimentally observed incremental length control but it leaves open the molecular origin of the initiator factor. According to Robert, the factor could be DnaA but as pointed out by the author, several experimental findings argue against realization of this essential control mechanism through this single factor alone.

## Concluding remarks

At the phenomenological level, “the facts on the ground” are mostly established now, yet the molecular details of how the coupling between nucleoid replication, cell growth, and division are realized remain to be clarified. Localized molecular interactions are playing major roles in these couplings, but large-scale global dynamics resulting from entropic forces, phase separation, self-assembly, and pattern formation are as important. An interdisciplinary approach is necessary to link all these processes into a coherent understanding. We hope that this e-book will spur further research in this fundamental area of biology and foster new collaborations between cell biologists, chemists, and physicists. A follow-up series of articles on this field is likely to arise as new results emerge in future studies.

## Author contributions

All authors listed contributed to the writing of this article, and approved it for publication.

### Conflict of interest statement

The authors declare that the research was conducted in the absence of any commercial or financial relationships that could be construed as a potential conflict of interest.

